# Low Tidal Volume Ventilation Is Poorly Implemented for Patients in North American and United Kingdom ICUs Using Electronic Health Records

**DOI:** 10.1016/j.chest.2023.09.021

**Published:** 2023-09-27

**Authors:** Romit J. Samanta, Ari Ercole, Steven Harris, Charlotte Summers

**Affiliations:** a Victor Phillip Dahdaleh Heart & Lung Research Institute, University of Cambridge, Cambridge, England; b Centre for AI in Medicine, University of Cambridge, Cambridge, England; c Bloomsbury Institute of Intensive Care Medicine, University College London, London, England

**Keywords:** acute hypoxemic respiratory failure, electronic health records, low tidal volume ventilation, outcomes

## Abstract

**Background:**

Low tidal volume ventilation (LTVV; < 8 mL/kg predicted body weight [PBW]) is a well-established standard of care associated with improved outcomes. This study used data collated in multicenter electronic health record ICU databases from the United Kingdom and the United States to analyze the use of LTVV in routine clinical practice.

**Research Question:**

What factors are associated with adherence to LTVV in the United Kingdom and North America?

**Study Design:**

This was a retrospective, multicenter study across the United Kingdom and United States of patients who were mechanically ventilated.

**Methods:**

Factors associated with adherence to LTVV were assessed in all patients in both databases who were mechanically ventilated for > 48 h. We observed trends over time and investigated whether LTVV was associated with patient outcomes (30-day mortality and duration of ventilation) and identified strategies to improve adherence to LTVV.

**Results:**

A total of 5,466 (Critical Care Health Informatics Collaborative [CCHIC]) and 7,384 electronic ICU collaborative research database [eICU-CRD] patients were ventilated for > 48 h and had data of suitable quality for analysis. The median tidal volume (V_T_) values were 7.48 mL/kg PBW (CCHIC) and 7.91 mL/kg PBW (eICU-CRD). The patients at highest risk of not receiving LTVV were shorter than 160 cm (CCHIC) and 165 cm (eICU-CRD). Those with BMI > 30 kg/m^2^ (CCHIC OR, 1.9 [95% CI, 1.7-2.13]; eICU-CRD OR, 1.61 [95% CI, 1.49-1.75]) and female patients (CCHIC OR, 2.39 [95% CI, 2.16-2.65]; eICU-CRD OR, 2.29 [95% CI, 2.26-2.31]) were at increased risk of having median V_T_ > 8 mL/kg PBW. Patients with median V_T_ < 8 mL/kg PBW had decreased 30-day mortality in the CCHIC database (CCHIC cause-specific hazard ratio, 0.86 [95% CI, 0.76-0.97]; eICU-CRD cause-specific hazard ratio, 0.9 [95% CI, 0.86-1.00]). There was a significant reduction in V_T_ over time in the CCHIC database.

**Interpretation:**

There has been limited implementation of LTVV in routine clinical practice in the United Kingdom and the United States. V_T_ > 8 mL/kg PBW was associated with worse patient outcomes.

FOR EDITORIAL COMMENT, SEE PAGE 233

**Take-home Points**

**Study Question:** What were the factors associated with adherence to low tidal volume ventilation in the United Kingdom and North America?

**Results:** Shorter patients (most often female) and those with BMI > 30 kg/m^2^ were most at risk of not receiving low tidal volume ventilation, and initial ventilator settings were often not altered even in the context of worsened respiratory failure.

**Interpretation:** There has been limited implementation of low tidal volume ventilation in routine clinical practice in the United Kingdom and the United States, and tidal volume > 8 mL/kg predicted body weight was associated with worse patient outcomes.

Low tidal volume ventilation (LTVV) remains one of the few interventions in patients with ARDS that has been consistently shown to improve patient mortality.^1^, ^2^, ^3^ LTVV has been intensively studied, and benefits have been reported in populations of mechanically ventilated patients with and without ARDS; its implementation is widely recommended.^4^, ^5^, ^6^, ^7^ Despite LTVV being a cost-effective intervention, implementation remains variable across institutions, which has been shown to affect patient outcomes.^8^, ^9^, ^10^

The availability of pooled, anonymized electronic health records offers the opportunity to examine routine clinical practice across multiple centers and longitudinally. We investigated the adherence to LTVV in multiple centers in a retrospective observational study using pooled data from intensive care electronic health records in the United Kingdom and United States.^11^^,^^12^ The aim of the current study was to determine the factors associated with implementation of LTVV and whether these factors were consistent across the United Kingdom and the United States. We hypothesized that baseline clinical features could identify patients who were at risk of not receiving LTVV, and failure to implement LTVV was associated with adverse patient outcomes in routine clinical practice.

## Study Design and Methods

### Study Design and Data Sources

This retrospective, multicenter analysis included ventilated ICU patients whose data were recorded in one of two databases: one from the United Kingdom and the other from the United States. The UK data were from 11 ICUs from five academic centers (Critical Care Health Informatics Collaborative [CCHIC]) that included 47,391 patient episodes. The US data were obtained from the electronic-ICU collaborative research database (eICU-CRD) and were collected from academic and nonacademic medical centers that used the tele-ICU program called eICU (electronic ICU, Philips Healthcare), which included 200,859 patient episodes. Ethical approvals and data governance details are included in e-Appendix 1.

### Inclusion and Exclusion Criteria

The study included all adult (aged > 16 years) intubated patients who had undergone a period of invasive mechanical ventilation and had corresponding arterial blood gas, ventilation (tidal volume [V_T_] and positive end-expiratory pressure), height, and sex data. Patient height and sex were used to calculate predicted body weight (PBW).^1^

Patients who were ventilated for < 48 h and those who died within 48 h of ICU admission were excluded. Patients with a height < 1.2 m were excluded due to the potential inaccuracy of the PBW formulae and the nonlinear relationship between anatomical dead space and height.^13^

### Outcomes

The primary outcome was adherence to LTVV and identification of the factors associated with adherence in a multicenter setting. Secondary outcomes included the association between LTVV and all-cause ICU mortality and duration of mechanical ventilation, changes in V_T_ over time (2014 to 2019), and adherence to LTVV during periods of acute hypoxemic respiratory failure (AHRF).

### Defining AHRF

We assessed the management of ventilated patients with AHRF, defined as a Pao_2_:Fio_2_ ratio < 300 mm Hg while receiving positive end-expiratory pressure ≥ 5 cm H_2_O and Fio_2_ ≥ 0.4. The Pao_2_:Fio_2_ ratios were calculated only for those patients receiving Fio_2_ ≥ 0.4 due to concerns regarding the utility of this measure at low Fio_2_.^14^ Mild, moderate, and severe AHRF were defined according to Berlin ARDS definition thresholds for Pao_2_:Fio_2_ if there were two corroborating blood gas analyses within a 12 h window, an approach used in contemporaneous ARDS studies.^15^ A 6 h window following a qualifying blood gas finding was captured, and the median V_T_ from this period was reported. Because of the anonymization process, we were unable to determine whether patients had been diagnosed with ARDS.

### Ventilation Periods and Parameters

Values from periods of noninvasive ventilation were excluded. Ventilatory data were available across the databases at differing measurement frequencies, with some recorded at minute-by-minute intervals and others hourly or less frequently. To unify our analyses across both databases, ventilatory data were abstracted at 1 h intervals using the median value for multiple observations in 1 h. New episodes of ventilation were demarcated by pauses in V_T_ recordings that were > 24 h. Missing values were not imputed.

Mechanical ventilation modes were not available in the CCHIC database and were sparsely recorded in the eICU-CRD. We restricted our analyses to periods of controlled ventilation determined by the set and observed ventilator rates, allowing for a 15% difference between these values to determine periods that were likely to be controlled. For example, if the ventilator rate was set to 12 breaths per minute, we would allow up to 14 breaths per minute to define the given period as controlled mode ventilation. Because plateau pressures were not recorded in the CCHIC database, inferences relating to driving pressure or static compliance could not be made.

To identify opportunities to improve practice, we examined the V_T_ during the first 6 h, 24 h (day 1), between 24 and 48 h (day 2), and during periods of the worst AHRF to determine if initial settings were changed over time, or whether there was a persistence of initial practice. Changes in V_T_ were mapped by using Sankey diagrams.

The optimum starting V_T_ was calculated (in milliliters) for patients using cumulative distribution curves based on the heights of patients who were mechanically ventilated in each database. The aim was to reduce the rate of patients receiving > 8 mL/kg PBW to < 10% of patients.

### Statistical Methods

Descriptive statistics are reported as median with interquartile range, mean with SD, and counts with percentages. When comparing subgroups of patients, we used ORs, *t* tests for normally distributed data, and Wilcoxon rank sum tests for non-normally distributed data. ICU mortality, LTVV use, and duration of ventilation were evaluated by using generalized mixed linear models, attributing random effects to the hospital identifier. The interclass correlation coefficient was used to report the explained variance from random effects. Ventilation duration was analyzed by using a log-linked Poisson distribution, and results are reported as the relative risk ratios (RRRs). Because data from some hospitals were sparse, Markov chain Monte Carlo methods with no U-turn sampling (RStan, 1,000 iterations, 500 warmups, two chains) were used to ensure model convergence and check the results from the generalized linear mixed effects models.

Age, sex, height, ethnicity, Acute Physiology and Chronic Health Evaluation (APACHE) scores (APACHE II in CCHIC, APACHE IV in eICU-CRD), and type of admission (medical/surgical) were used as covariates as these were consistently measured and relatively complete in both databases. To assess the interactions between covariates, log odds (logit) plots were used. The effect sizes and statistical significance of interaction terms were checked and the threshold values for effects calculated. To aid interpretability, generalized linear models with random effects were not fitted with interactions. To ensure that we were not inadvertently unmasking the effects of closely related baseline variables (sex and height) when estimating the primary outcome, we performed a separate subgroup analysis of patients stratified according to sex. ORs were expressed as point estimates with 95% confidence limits, and the threshold *P* value for significance was < .05.

The change in practice over time was examined in the CCHIC database by calculating the median V_T_ for a given patient, weighted by duration of ventilation in each month. A linear model for weighted median V_T_ against time was used to estimate this change, which was expressed as milliliters per kilogram PBW per quarter (3-month period). Results were visualized by using a locally estimated scatterplot smoothing curve.

To determine the association between LTVV and patient outcomes, a competing risk model was used to estimate the subdistribution hazards for patients who remained ventilated, had died, or had been extubated 30 days following initiation of invasive ventilation. Adjustments were made for age and APACHE score. These were described using cumulative incidence function curves with censoring at day 30.

Data queries were performed with PgAdmin 4 (version 3.0, PostgreSQL Global Development Group) and R version 3.6.2 (R Core Team, R Foundation for Statistical Computing). Data curation codes for the eICU-CRD are included in e-Appendix 2. Given that the two databases differed in terms of their respective time periods covered, populations, data quality, and size, direct comparisons between them were not made.

## Results

**Figure 1:**
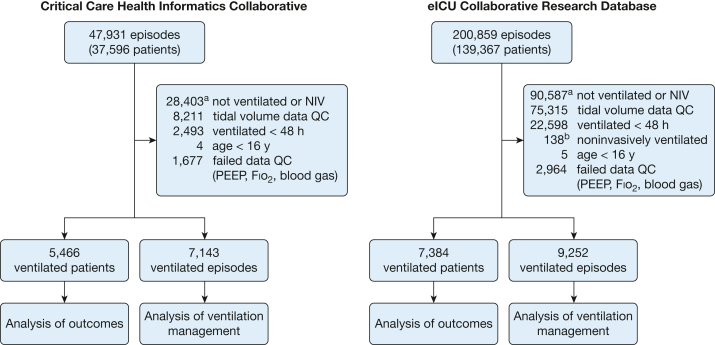
Study flow diagram outlining data cleaning steps for each database and the numbers included in the analysis. ^a^Values in these boxes refer to patient episodes, not unique patients. ^b^Some patients were identified as having periods of both invasive ventilation and NIV. These patients were excluded to avoid misattributing measured volumes during NIV periods. NIV = noninvasive ventilation; PEEP = positive end-expiratory pressure; QC = quality control.

The CCHIC and eICU-CRD databases contained data on 47,931 and 200,859 patient episodes, respectively, approximately one-half of which (CCHIC, 48.1%; eICU-CRD, 54.9%) involved a period of invasive mechanical ventilation. Following data curation, 5,466 and 7,384 patients were identified who were ventilated for > 48 h and had data suitable for analysis (Fig 1). Patients were ventilated for a median duration of 6.9 days in CCHIC (95% CI, 3.6-14.9) and 5.8 days in eICU-CRD (95% CI, 3.5-10.3) (Table 1).

### Factors Associated With Adherence to LTVV

**Figure 2:**
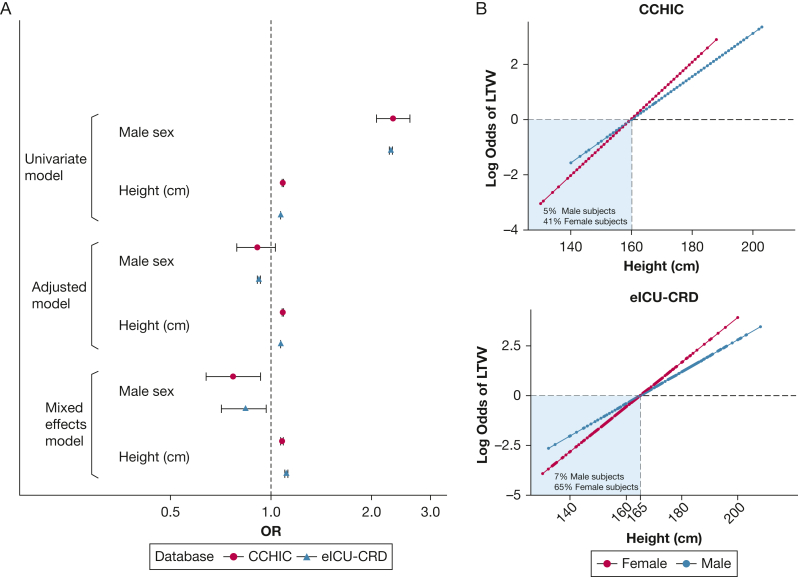
A, B, Analysis of factors predicting whether patients received LTVV. A, The forest plot shows the odds of patient receiving LTVV based on sex or height as univariate analysis or if adjusted for other covariates: age, ethnicity, Acute Physiology and Chronic Health Evaluation score, admission type, and hospital location as a random effect (other effect sizes not shown for clarity [Table 3]). Male patients were more likely to receive LTVV (unadjusted model) but not if their height was considered. Taller patients were consistently associated with receiving LTVV across all models regardless of their sex with consistent effect sizes. B, Interaction between height and sex on the log odds of receiving LTVV for each database. If these lines were parallel, this would have suggested that there was no interaction between height and sex. The lines happen to intersect each other, and the log odds equals zero line (dashed line, corresponding to an OR of 1) at 160 cm and 165 cm in the CCHIC/eICU-CRD databases, respectively. These values can be interpreted as the threshold height at which both female and male patients were likely to receive LTVV. Ventilated female patients shorter than these heights were less likely to receive LTVV than male patients of the same height. CCHIC = Critical Care Health Informatics Collaborative; eICU-CRD = electronic ICU collaborative research database; LTVV = low tidal volume ventilation.

We found that taller patients, following adjustment for sex, age, patient type (medical or surgical), APACHE score, and treating hospital, had a higher likelihood of receiving LTVV in both databases (Fig 2A, Table 2). A 10 cm height increment corresponded to an increased probability of LTVV in both the CCHIC (OR, 1.08; 95% CI, 1.07-1.09) and eICU-CRD (OR, 1.08; 95% CI, 1.08-1.09) databases (Table 2) and a reduction in median V_T_ per ventilation episode by 0.27 mL/kg PBW.

**Table 1:** Characteristics of Ventilated Patients Within Each Database

Characteristic	CCHIC		eICU-CRD
Demographic characteristics			
No. of hospitals	5		107
No. of ventilation episodes	7,143		9,252
Teaching hospital^a^	100%		44.8%
No. of unique patients	5,466		7,384
Male	3,374 (62%)		4,181 (57%)
Age, median (IQR), y	61 (48-72)		63 (52-72)^b^
Ethnicity			
White	4,051 (74.1%)	Caucasian	5,760 (78%)
Other	801 (14.7%)	African American	894 (12.1%)
Black	358 (6.6%)	Hispanic	203 (2.8%)
Asian^c^	194 (3.6%)	Asian	88 (1.2%)
Chinese	32 (0.6%)	Native American	54 (0.8%)
Mixed race	29 (0.5%)	Other/unknown	275 (3.7%)
Not stated	1 (< 0.1%)	…^d^	…^d^
BMI, mean ± SD, kg/m^2^	26.9 ± 6.8		30.0 ± 9.9
BMI group			
< 18.5 kg/m^2^	296 (5.4%)		481 (6.5%)
18.5-25 kg/m^2^	2,268 (41.5%)		1,912 (25.9%)
25-30 kg/m^2^	1,554 (28.4%)		1,869 (25.3%)
30-35 kg/m^2^	785 (14.4%)		1,363 (18.5%)
35-40 kg/m^2^	311 (5.7%)		774 (10.5%)
> 40 kg/m^2^	252 (4.6%)		972 (13.2%)
Admission type			
Emergency (local)	3,731 (68.3%)	ED/floor/cardiac center	4,406 (60.0%)
Emergency (transfer)	619 (11.3%)	OR/PACU/recovery	1,148 (15.5%)
Planned	732 (13.4%)	Direct admit	977 (8.9%)
Repatriation	32 (0.6%)	Other ICU/step down unit	382 (5.2%)
		Other hospital	692 (9.4%)
Primary admission diagnosis			
Surgical	1,202 (22.0%)		1,211 (11.1%)
GI	398		317
Cardiothoracic	252		372
Neurosurgical	196		159
Trauma	180		185
Vascular	75		82
Genitourinary	52		28
Orthopedic/plastic surgery	41		46
Other	8		22
Medical	4,247 (77.7%)		6,155 (66.5%)
Respiratory	1,970		1,584
Cardiovascular (including sepsis)	868		2,686
Neurologic	457		938
GI	345		275
Trauma (nonsurgical)	245		465
Genitourinary	150		74
Metabolic/endocrine	95		71
Hematologic/immunologic	92		19
Poisoning	78		…^d^
Other	60		43
Comorbidities^e^			
CPR prior to ICU admission	563 (10.3%)		1,379 (18.7%)
Cardiovascular	877		4,552
Hypertension	623 (71%)		1,795 (39.4%)
Arrhythmia	112 (12.8%)		540 (12.5%)
Ischemic heart disease	43 (4.9%)		429 (9.4%)
Congestive cardiac failure	36 (4.1%)		461 (10.1%)
Endocrine	511		2,534
Diabetes	441 (86.3%)		1,721 (67.9%)
Psychiatric	392		…^d^
Alcohol dependence	179 (45.7%)		…^d^
Respiratory	368		2,060
COPD	277 (75.3%)		749 (36.4%)
Home oxygen	…^d^		216 (10.5%)
Neurologic	54		1,328
Stroke	3 (5.6%)		412 (31.1%)
Dementia	…^d^		165 (82.1%)
Renal	137		1,706
Chronic kidney disease	98 (71.5%)		622 (90.7%)
Hematology/oncology	93		979
malignancy	46 (49.5%)		871 (89%)
GI	43		473
Cirrhosis	11 (25.6%)		377 (79.8%)
APACHE II score, mean ± SD	18.5 ± 6.6		…^d^
APACHE IV score, mean ± SD	…^d^		81.6 ± 29.5
Outcomes			
ICU mortality	1,151 (28.4%)		1,466 (19.9%)
Hospital mortality	1,835 (33.6%)		2,106 (28.5%)
Duration of invasive ventilation,^f^ median (IQR), d	6.9 (3.6-14.9)		5.8 (3.5-10.3)
Length of ICU stay,^f^ median (IQR), d	13.4 (7.7-23.9)		8.9 (5.6-14.2)
Length of hospital stay,^g^ median (IQR), d	32 (18-58)		14 (8.6-22)
Hospital discharge destination^g^			
Home	1,997 (55.2%)	Home	1,751 (33.2%)
Rehabilitation	169 (4.7%)	Skilled nursing facility	1,332 (25.2%)
Nursing home	65 (1.8%)	Other hospital	543 (10.3%)
Other health institution	21 (0.6%)	Rehabilitation	701 (13.3%)
Hospice	15 (0.4%)	Nursing home	75 (1.42%)
Other facility	8 (0.2%)	Other facility	782 (14.8%)
Unknown/missing	1,346 (37.1%)	Unknown/missing	94 (1.8%)

APACHE = Acute Physiology and Chronic Health Evaluation; CCHIC = Critical Care Health Informatics Collaborative; eICU-CRD = eICU collaborative research database; IQR = interquartile range; NA = not available/missing data; OR = operating room; PACU = postanesthesia care unit.

a In the United Kingdom, teaching hospital refers to an academic health center.

b Ages > 89 years are considered potentially identifying data by the Health Insurance Portability and Accountability Act of 1996 and thus are coded “>89.” We have presumed an age of 90 years in these patients and used the median value to describe the population average.

c In the United Kingdom, “Asian” ethnicity refers to those who identify as originating from South Asia/the Indian subcontinent.

d Missing data are due to differences in how patient features were coded in each of the two databases.

e Percentages for comorbidities apply to the number of patients within each organ system category, not the number of patients in total. Many patients had multiple comorbid conditions.

f ICU survivors.

g Hospital survivors.

**Table 2:** Factors Associated With Administration of Low Tidal Volume Ventilation

Variable	CCHIC				eICU-CRD			
	Estimate	SE	OR (95% CI)	*P*	Estimate	SE	OR (95% CI)	*P*
Fixed effects								
Sex: male	–0.25	0.09	0.78 (0.65-0.94)	.008	–0.093	0.008	0.91 (0.90-0.93)	< .001
Height (cm)	–0.075	0.005	1.08 (1.07-1.09)	< .001	0.077	0.00^a^	1.08 (1.08-1.08)	< .001
Age	–0.003	0.002	1.00 (0.99-1)	.12	0.003	0.00^a^	1.003 (1.00-1.00)	< .001
APACHE score^b^	–0.00^a^	0.006	1.00 (0.98-1.01)	.92	–0.001	0.001	1.00 (0.99-1.00)	< .001
Ethnicity: non-White/non-Caucasian^c^	0.49	0.08	1.63 (1.38-1.92)	< .001	0.28	0.008	1.33 (1.30-1.35)	< .001
Admission type: medical	0.35	0.1	1.42 (1.17-1.72)	< .001	0.39	0.01	1.48 (1.45-1.50)	< .001
Random effect	Variance	SD	ICC		Variance	SD	ICC	
Treating hospital	0.02	0.14	0.01	…	1.01	1.005	0.23	…

Effect sizes were estimated by using a mixed effects logistic regression model. APACHE = Acute Physiology and Chronic Health Evaluation; CCHIC = Critical Care Health Informatics Collaborative; eICU-CRD = electronic ICU collaborative research database; ICC = inter-class correlation (a measure of the proportion of the variance explained by the grouping structure, which in this case is the treating hospital).

a Values < 0.0001 have been abbreviated to 0.00 for display in the table.

b APACHE II score was used for CCHIC, APACHE IV for eICU-CRD.

c Reference groups were “White/White British” (CCHIC) or “Caucasian” (eICU-CRD).

Our logistic regression interaction model showed that the critical heights associated with increased likelihood of LTVV administration were > 160 cm (CCHIC) and > 165 cm (eICU-CRD) (Fig 2B); 41% and 62.1% of ventilated female patients (CCHIC/eICU-CRD, respectively) had heights below these thresholds. Conversely, only 5% and 7.6% of ventilated male participants (CCHIC/eICU-CRD) were below this threshold.

A primary admission diagnosis that was medical in nature was associated with an increased likelihood of receiving LTVV, even when adjustments for an interaction between height and sex were considered: OR for LTVV 1.18 (95% CI, 1.02-1.36, *P* = .02) in CCHIC and 1.3 (95% CI, 1.13-1.49, *P* < .0001) for eICU-CRD (Table 2).

**Figure 3:**
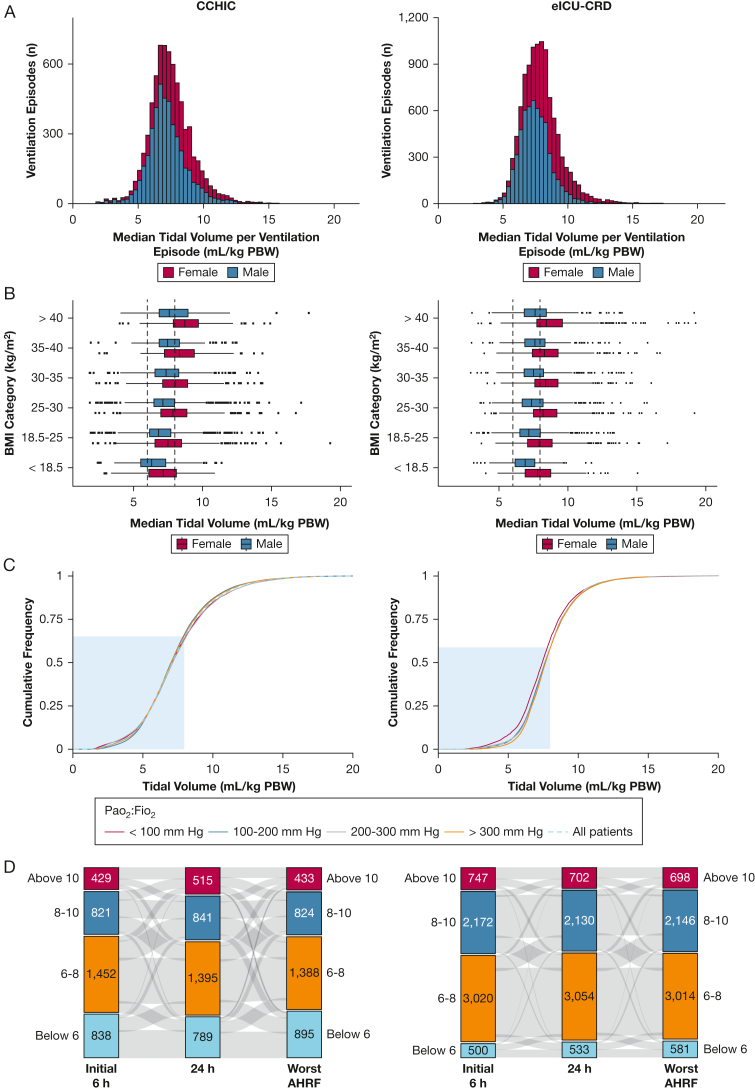
A-D, Analysis of tidal volume management in different patient groups. The median tidal volume for each patient’s ventilation period was calculated and converted to milliliters per kilogram by using the PBW formula of the National Heart, Lung, and Blood Institute-funded Acute Respiratory Distress Syndrome Clinical Trial Network. A, Female patients who were mechanically ventilated consistently received higher tidal volume, on average, than male patients who were mechanically ventilated. B, This was more apparent in female patients with higher BMIs and was observed in both databases. C, There appeared to be no change in tidal volume, in either database, for patients with different grades of acute hypoxemic respiratory failure. These grades were determined based on consistent Pao_2_:Fio_2_ ratios in a 12 h window, while receiving Fio_2_ ≥ 40% and ≥ 5 cm H_2_O positive end-expiratory pressure. D, There was little variation in tidal volume for patients, at 24 h following admission or when patients experienced their worst period of acute hypoxemic respiratory failure following this 24 h period. CCHIC = Critical Care Health Informatics Collaborative; eICU-CRD = electronic ICU collaborative research database; PBW = predicted body weight.

The failure to adjust V_T_ for PBW led to female patients being consistently ventilated with significantly higher V_T_ than male patients across all time points (Fig 3A, Table 3), all BMI groups (Fig 3B), and all severities of AHRF (Table 3) in both databases. In addition, the likelihood of not receiving LTVV was consistently higher for female patients (CCHIC OR, 2.39 [95% CI, 2.16-2.65]; eICU-CRD OR, 2.29 [95% CI, 2.26-2.31]) and those with BMI > 30 kg/m^2^ (CCHIC OR, 1.9 [95% CI, 1.7-2.13]; eICU-CRD OR, 1.61 [95% CI, 1.49-1.75]). Following adjustments for patient age and type of admission, we found that patient sex was not significantly associated with the cause-specific risk of 30-day mortality (e-Table 1).

**Table 3:** Tidal Volumes During Invasive Mechanical Ventilation

Variable	CCHIC		eICU-CRD	
Tidal volume, mL/kg PBW				
All periods	7.48 ± 1.69		7.91 ±1.53	
Day 1	7.39 ± 1.95		7.98 ± 1.63	
Day 2	7.62 ± 2.12		8.04 ± 1.88	
Worst Pao_2_:Fio_2_	7.42 ± 2.66		8.01 ± 1.99	
By sex	Male	Female	Male	Female
All periods	7.22 ± 1.55	7.89 ± 1.82	7.51 ± 1.31	8.41 ± 1.64
Day 1	7.10 ± 1.78	7.87 ± 2.11	7.54 ± 1.35	8.55 ± 1.76
Day 2	7.33 ± 1.98	8.07 ± 2.24	7.61 ± 1.66	8.57 ± 2.01
Worst Pao_2_:Fio_2_	7.19 ± 2.56	7.79 ± 2.81	7.57 ± 1.74	8.58 ± 2.14
Median tidal volume/episode < 8 mL/kg PBW (%)	33.9		43.5	
	Male	Female	Male	Female
	29.4	40.5	41.6	58.4
Tidal volume during AHRF periods, mL/kg PBW				
Patients with AHRF	3,249 (59.4%)		3,627 (39.2%)	
Pao_2_:Fio_2_ < 100, mm Hg	7.24 ± 2.42		7.98 ± 1.98	
Pao_2_:Fio_2_ 100-200, mm Hg	7.40 ± 2.15		8.01 ± 1.84	
Pao_2_:Fio_2_ 200-300, mm Hg	7.56 ± 2.02		8.00 ± 1.86	
By sex	Male	Female	Male	Female
Pao_2_:Fio_2_ < 100, mm Hg	7.07 ± 2.31	7.54 ± 2.57	7.64 ± 1.71	8.49 ± 2.22
Pao_2_:Fio_2_ 100-200, mm Hg	7.21 ± 2.00	7.74 ± 2.35	7.67 ± 1.67	8.49 ± 1.97
Pao_2_:Fio_2_ 200-300, mm Hg	7.35 ± 1.88	7.91 ± 2.2	7.68 ± 1.8	8.48 ± 1.85
Tidal volume by BMI group, mL/kg PBW				
< 18.5 kg/m^2^	6.77 ± 154		7.41 ± 1.42	
18-25 kg/m^2^	7.24 ± 1.59		7.65 ± 1.43	
25-30 kg/m^2^	7.55 ± 1.63		7.89 ± 1.45	
30-35 kg/m^2^	7.71 ± 1.63		8.03 ± 1.42	
35-40 kg/m^2^	7.99 ± 1.71		8.09 ± 1.58	
> 40 kg/m^2^	8.66 ± 2.33		8.38 ± 1.84	
By sex	Male	Female	Male	Female
< 18.5 kg/m^2^	6.49 ± 1.49	7.09 ± 1.54	6.95 ± 1.17	7.90 ± 1.51
18-25 kg/m^2^	7.02 ± 1.49	7.61 ± 1.68	7.33 ± 1.21	8.11 ± 1.91
25-30 kg/m^2^	7.31 ± 1.54	7.97 ± 1.70	7.51 ± 1.51	8.47 ± 1.55
30-35 kg/m^2^	7.49 ± 1.53	8.11 ± 1.73	7.67 ± 1.27	8.55 ± 1.46
35-40 kg/m^2^	7.69 ± 1.51	8.41 ± 1.89	7.69 ± 1.34	8.51 ± 1.70
> 40 kg/m^2^	8.04 ± 1.86	9.15 ± 2.54	7.86 ± 1.62	8.81 ± 1.78

Data are presented as mean ± SD unless indicated otherwise. All tidal volumes are in milliliters per kilogram (PBW), which was calculated by using the National Heart, Lung, and Blood Institute-funded Acute Respiratory Distress Syndrome Clinical Trial Network formula. AHRF = acute hypoxemic respiratory failure (Pao_2_:Fio_2_ < 300 mmHg on FiO_2_ ≥ 0.4 with ≥5 cmH_2_O positive end-expiratory pressure); CCHIC = Critical Care Health Informatics Collaborative; eICU-CRD = electronic ICU collaborative research database; PBW = predicted body weight.

### LTVV Adherence During Periods of AHRF

A total of 3,249 (59.4%, CCHIC) and 3,627 (39.2%, eICU-CRD) ventilated patients in each database had blood gas analyses consistent with AHRF (Pao_2_:Fio_2_ < 300 mm Hg with > 5 cm H_2_O positive end-expiratory pressure and Fio_2_ > 0.4). Of these, 760 (13.9%, CCHIC) and 1,360 (18.4%, eICU-CRD) had a Pao_2_:Fio_2_ ratio < 100 mm Hg (Table 3).

During periods of AHRF, 34.8% (CCHIC) and 39.7% (eICU-CRD) of patients received V_T_ > 8 mL/kg PBW (Table 3). For periods in which Pao_2_:Fio_2_ fell below 100 mm Hg, 35.3% (CCHIC) and 35.9% (eICU-CRD) of patients did not receive LTVV. The median V_T_ during these periods were 7.2 and 8.0 mL/kg PBW (CCHIC/eICU-CRD).

There was a general failure to adjust V_T_ in response to deterioration in oxygenation status (Figs 3C, 3D). Within a 6 h window following each patient’s worst recorded Pao_2_:Fio_2_ value, relative proportions of V_T_ > 8 mL/kg PBW decreased by 14.9% in the CCHIC database and slightly increased by 0.4% in the eICU-CRD database.

### LTVV Adherence at Different Times During Patients’ Ventilation Episodes

Little variation in V_T_ was observed over the course of individual ventilation episodes. In the CCHIC database at 24 h, there was a 12.9% decrease in the proportion of V_T_ that was consistent with LTVV compared with the initial 6 h period. However, in the eICU-CRD, there was a slight increase in the adoption of LTVV at 24 h (0.8%), compared with the first 6 h (Fig 3D).

### Secondary Outcomes

**Figure 4:**
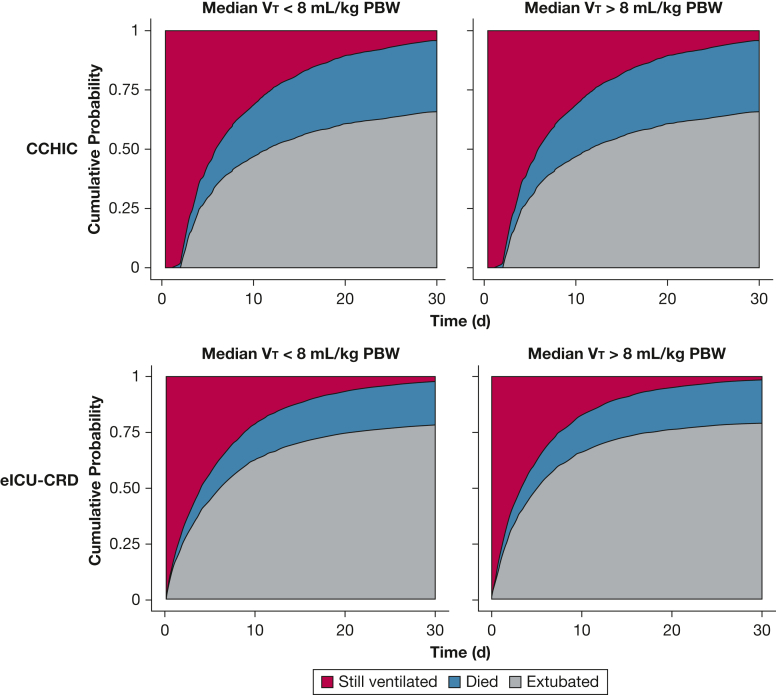
Cumulative incidence plots for patients with a median V_T_ above or below 8 mL/kg PBW showing the relative incidence of each competing outcome (death, still ventilated, extubated) at day 30 after starting ventilation. The cause-specific hazard ratio for death at 30 days was lower in patients receiving LTVV in the CCHIC database (SHR, 0.86; 95% CI 0.76-0.97; *P* < .001) but not in the eICU-CRD database (SHR, 0.9 [95% CI, 0.81-1.01; *P* = .06]). LTVV was, however, associated with a lower probability of extubation by day 30 in both databases (CCHIC SHR, 0.69 [95% CI, 0.64-0.75, *P* < .001]; eICU-CRD SHR, 0.88 [95% CI, 0.83-0.92, *P* < .001]). CCHIC = Critical Care Health Informatics Collaborative; eICU-CRD = electronic ICU collaborative research database; PBW = predicted body weight; SHR = cause-specific hazard ratio; V_T_ = tidal volume.

LTVV, with adjustment for patient age and APACHE score, was associated with an improved cause-specific hazard ratio (SHR) of death for patients in the CCHIC database (SHR, 0.86; 95% CI, 0.76-0.97; *P* < .001) (Table 4). This association did not cross the threshold for significance in the eICU-CRD (SHR, 0.9; 95% CI, 0.81-1.0; *P* = .06). Patients who received LTVV were more likely to remain ventilated at day 30 in both databases: CCHIC SHR, 0.69 (95% CI 0.64-0.75, *P* < .001); eICU-CRD SHR, 0.88 (95% CI 0.82-0.92, *P* < .001) (Fig 4). They also had a longer duration of ventilation in those surviving to ICU discharge (CCHIC RRR, 1.39 [95% CI, 1.34-1.42]; eICU-CRD RRR, 1.10 [95% CI 1.08-1.12]) (Table 5).

**Table 4:** Effect of LTVV on Outcomes of Patients Mechanically Ventilated for > 48 h From Both Databases

Outcome	CCHIC		eICU-CRD	
	SHR (95% CI)	*P*	SHR (95% CI)	*P*
Death				
LTVV	0.86 (0.76-0.97)	< .001	0.9 (0.81-1.00)	.06
Age	1.01 (0.99-1.01)	< .001	1.01 (1.01-1.01)	< .001
APACHE	1.06 (1.05-1.07)	< .001	1.02 (1.01-1.02)	< .001
Extubation				
LTVV	0.69 (0.64-0.75)	< .001	0.88 (0.83-0.92)	< .001
Age	1.00 (0.99-1.00)	.7	1.003 (1.001-1.005)	< .001
APACHE	0.95 (0.94-0.96)	< .001	0.997 (0.996-0.998)	< .001

Outcomes at day 30 were assessed by using competing outcomes and expressed as SHRs. For example, with respect to the outcome of being extubated by day 30, death was used as a competing hazard. Although LTVV was associated with a lower cause-specific hazard of death by day 30, patients managed in this way were not more likely to be extubated. APACHE = Acute Physiology and Chronic Health Evaluation; CCHIC = Critical Care Health Informatics Collaborative; eICU-CRD = electronic ICU collaborative research database; LTVV = low tidal volume ventilation; PBW = predicted body weight; SHR = cause-specific hazard ratio.

### Temporal Trends Show a Reduction in V_T_

**Figure 5:**
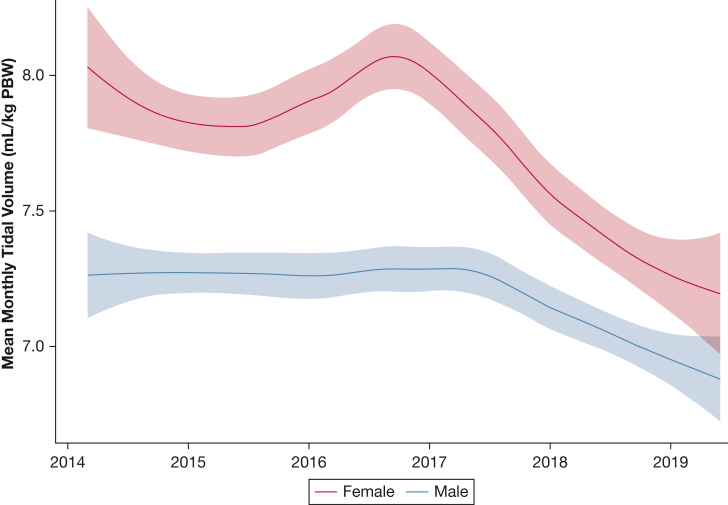
Trends (locally estimated scatterplot smoothing) in administered tidal volume over time, for both male and female patients in the Critical Care Health Informatics Collaborative database. The median tidal volume for each patient’s ventilation period was calculated and aggregated with all the other mechanically ventilated patients that month using a weighted mean. A locally estimated scatterplot smoothing curve was fitted to the time series to show changes in practices over time with bandwidth that included 65% of local points. Following December 2016, there was a consistent and significant decrease in median tidal volume per ventilation episode for both male patients (–0.19 mL/kg PBW per quarter; 95% CI, 0.08-0.29) and female patients (–0.26 mL/kg PBW per quarter; 95% CI, 0.07-0.46). PBW = predicted body weight.

There was an easily discernible trend in the CCHIC database, with a significant reduction in median V_T_ for both male (–0.19 mL/kg PBW per quarter; 95% CI, 0.08-0.29; *P* = .0015) and female patients (–0.26 mL/kg PBW per quarter; 95% CI, 0.07-0.46; *P* = .012) following December 2016 (Fig 5).

### Initial V_T_ Values and Strategies to Improve Practice

**Figure 6:**
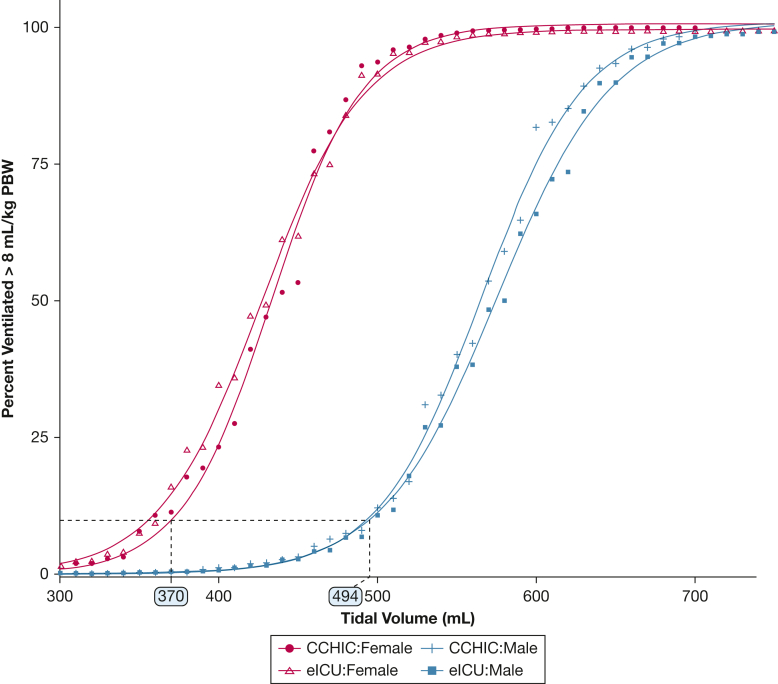
Cumulative density plot showing the percentage of patients who would be in receipt of tidal volume > 8 mL/kg PBW based on the height and sex of patients in each database. To reduce the risk of patients receiving > 8 mL/kg PBW to < 10% in the absence of the patient’s height, the initial tidal volume should be set to < 370 mL for female patients and < 494 mL for male patients. CCHIC = Critical Care Health Informatics Collaborative; eICU-CRD = electronic ICU collaborative research database; PBW = predicted body weight.

**e-Figure 1:**
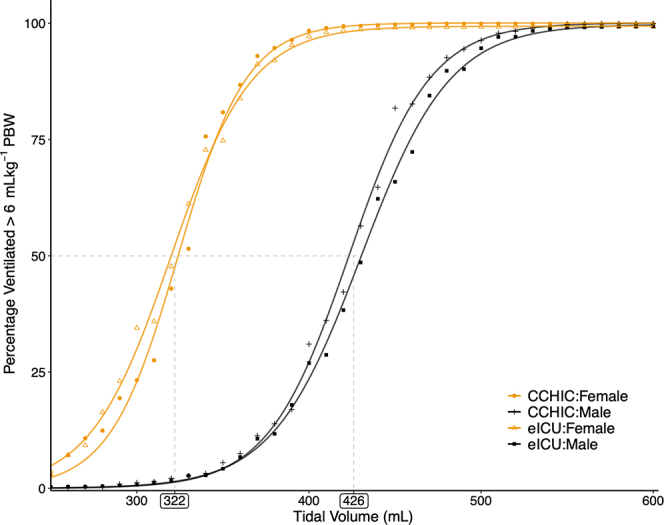


The median initial V_T_ (during the first 6 h of ventilation) for male patients was 492 mL and 545 mL in the CCHIC and eICU-CRD databases, respectively. For female patients, these values were 426 mL and 464 mL (CCHIC/eICU-CRD). This translated to > 42%/75% of female patients in the CCHIC/eICU-CRD databases receiving V_T_ > 8 mL/kg PBW from initiation of invasive mechanical ventilation. We calculated that to ensure > 90% of people received LTVV, initial V_T_ values of 370 mL for female and 494 mL for male patients should be used (Fig 6) if height-based adjustment of V_T_ was not undertaken. To ensure that < 50% of patients receive V_T_ > 6 mL/kg PBW, these values should be reduced to 320 mL for female and 430 mL for male patients (e-Fig 1).

## Discussion

Our analyses show the limited implementation of LTVV in routine clinical practice in a multicenter and international context. Despite the different environments, patient populations, ICU admission criteria, and health systems in the United Kingdom and United States, we found that the same factors were associated with implementation of LTVV. Patients of shorter stature were consistently exposed to higher V_T_ values, which resulted in female patients and patients with greater BMI being disproportionately at risk. We also found evidence that failure to implement LTVV increased the risk of 30-day mortality in the CCHIC cohort, showing the value of LTVV as standard of care for all patients. Because we used similar data curation statistical methods for both cohorts, we were able to determine the threshold heights for patients below which the risk of not receiving LTVV was increased for each population: 160 cm for CCHIC and 165 cm for eICU-CRD.

The median V_T_ values of patients in each database were similar (CCHIC, 7.48 mL/kg PBW; eICU-CRD, 7.91 mL/kg PBW) and consistent with other multicenter, observational data from ARDS (Large Observational Study to Understand the Global Impact of Severe Acute Respiratory Failure [LUNG-SAFE], 7.61 mL/kg PBW)^16^ and non-ARDS (Practice of Ventilation in Critically Ill Patients Without ARDS at Onset of Ventilation [ProVENT], 7.9 mL/kg PBW)^17^ populations. The proportion of patients who received LTVV was higher in eICU-CRD (43.5%) than in CCHIC (33.4%). Both values were consistent with the results from pooled analyses of non-ARDS patients in high-income countries (44.5%)^18^ and patients in the United Kingdom with ARDS (35.9%-38.5%).^19^ A similar discrepancy in the implementation of LTVV between European and non-European high-income countries has previously been described by Laffey et al.^20^

Another strength of the current study was the opportunity to observe the changes in longitudinal trends in V_T_ in a multicenter setting. The fall in V_T_ over time (Fig 5) shows that improvements in LTVV implementation are being adopted across institutions. Other groups have shown similar longitudinal trends but only in single-center studies or non-ICU patients.^21^, ^22^, ^23^

We noted a paucity of variation in V_T_ when comparing the initial values (first 6 h) vs the 24 h and 48 h V_T_, as well as during periods of the most severe hypoxemia within each ventilation episode. This result underscores the importance of a precise initial approach to mechanical ventilation, as we found poor practices tended to persist. Higher initial V_T_ has previously been associated with significantly increased mortality.^24^

The initiation of mechanical ventilation and admission to an ICU are often emergency situations, frequently occurring outside of normal working hours. Accurate height measurements, necessary for V_T_ titration, may not be readily available. To help address this issue, we calculated starting values in milliliters for patients in each study population. This may help mitigate the risk of exposing patients to harmful V_T_ in the absence of precise height data and help overcome other obstacles to implementation of LTVV.^25^^,^^26^ Interventions to enhance adherence to LTVV have been shown to be cost-effective.^8^ Our proposition offers a simple step that may help to highlight the importance of regular evaluation of V_T_.

Previous studies have shown disparities in LTVV implementation based on sex, with female patients less likely to receive LTVV. This has been associated with increased mortality in patients with ARDS.^27^ We did not observe a difference in mortality based on sex in either database (e-Table 1). This was consistent with the findings in other non-ARDS populations in whom similar differences in V_T_ between sexes have been described.^28^

We found that patients receiving LTVV were more likely to have a greater duration of ventilation. We hypothesized that these patients were those undergoing prolonged weaning (eg, with delayed respiratory or neurologic recovery) who may have self-regulated their V_T_ to their normal physiologic range of 6 to 8 mL/kg PBW or who may have had poor pulmonary compliance that required a prolonged period of lung-protective ventilation. The latter case could be an example of reverse causality that we could not address due to anonymization.

There are several important limitations to our findings that should be considered. Our study was a post hoc analysis, and due to data limitations, we were unable to accurately abstract ventilation modes to determine whether patients received controlled or spontaneous ventilatory modes. We were also unable to evaluate the effects of driving pressure or mechanical power on patient outcome because the required variables were not available in the databases. We chose not to impute missing data, as attempts to do so induced overrepresentation of high V_T_ in the imputed values.

The anonymization process meant that the absence of clinical notes prevented us from accounting for other reasons that might cause a high V_T_ to be recorded (eg, a drained pneumothorax).

Illness severity was reported using APACHE scores and were recorded reliably in both databases. However, APACHE scores do not evaluate organ dysfunction beyond the initial 24 h period. Because of missing data across each organ system domain, we could not reliably extract other illness severity scores (eg, Sequential Organ Failure Assessment) that may have captured peak illness severity more accurately.

## Interpretation

LTVV was poorly implemented across multiple ICUs in the United States and the United Kingdom. This was consistent with a failure to account for patient height when setting V_T_ values. This oversight was the principal contributory factor to female patients and those with higher BMI being exposed to higher V_T_. In the UK cohort, exposure to V_T_ > 8 mL/kg PBW was associated with increased risk of 30-day mortality.

**Table 5:** Association Between LTVV and Duration of Ventilation in Patients Ventilated for > 48 h

Variable	CCHIC			eICU-CRD		
	Estimate	SE	Relative Risk Ratio (95% CI)	Estimate	SE	Relative Risk Ratio (95% CI)
LTVV	0.33	0.01	1.39 (1.35-1.42)	0.10	0.001	1.10 (1.08-1.12)
Age	–0.001	0.00^a^	1.00 (1.00-1.00)	0.005	0.00^a^	1.00 (0.99-1.00)
Ethnicity: non-White/Caucasian^b^	–0.02	0.00^a^	0.87 (0.85-0.89)	0.07	0.01	1.08 (1.05-1.10)
APACHE score^c^	0.02	0.00^a^	1.02 (1.01-1.02)	0.00^a^	0.00^a^	1.00 (1.00-1.00)
Admission type: medical	0.15	0.02	1.17 (1.13-1.21)	–0.06	0.01	0.94 (0.92-0.97)

Length of stay data are modeled using the log-linked Poisson distribution. APACHE = Acute Physiology and Chronic Health Evaluation; CCHIC = Critical Care Health Informatics Collaborative; eICU-CRD = electronic ICU collaborative research database; LTVV = low tidal volume ventilation.

a Values < 0.0001 have been abbreviated to 0.00 for display in the table.

b Reference groups were “White/White British” (CCHIC) or “Caucasian” (eICU-CRD)

c APACHE-II score was used for CCHIC, APACHE-IV for eICU-CRD.

## Funding/Support

The CCHIC was funded by a grant from the 10.13039/501100000272National Institute for Health Research (NIHR). R. J. S. was an EMINENT Clinical Research Training Fellow (jointly funded by 10.13039/100004330GlaxoSmithKline plc and NIHR Cambridge 10.13039/100014461Biomedical Research Centre [BRC1215-20014]). C. S. was supported by the 10.13039/501100000265Medical Research Council [MR/P502091/1] and the NIHR Cambridge 10.13039/100014461Biomedical Research Centre [BRC1215-20014] while undertaking this research.

## Financial/Nonfinancial Disclosures

None declared.

## Supplementary Data

e-Online Data

Audio

## References

[1] Brower R.G., Matthay M.A., Acute Respiratory Distress Syndrome Network (2000). Ventilation with lower tidal volumes as compared with traditional tidal volumes for acute lung injury and the acute respiratory distress syndrome. N Engl J Med.

[2] Needham D.M., Colantuoni E., Mendez-Tellez P.A. (2012). Lung protective mechanical ventilation and two year survival in patients with acute lung injury: prospective cohort study. BMJ.

[3] Petrucci N., De Feo C. (2013). Lung protective ventilation strategy for the acute respiratory distress syndrome. Cochrane Database Syst Rev.

[4] Fan E., Del Sorbo L., Goligher E.C. (2017). An official American Thoracic Society/European Society of Intensive Care Medicine/Society of Critical Care Medicine Clinical Practice Guideline: mechanical ventilation in adult patients with acute respiratory distress syndrome. Am J Respir Crit Care Med.

[5] Gajic O., Dara S.I., Mendez J.L. (2004). Ventilator-associated lung injury in patients without acute lung injury at the onset of mechanical ventilation. Crit Care Med.

[6] Sutherasan Y., Vargas M., Pelosi P. (2014). Protective mechanical ventilation in the non-injured lung: review and meta-analysis. Crit Care.

[7] Serpa Neto A., Hemmes S.N.T., Barbas C.S.V. (2015). Protective versus conventional ventilation for surgery: a systematic review and individual patient data meta-analysis. Anesthesiology.

[8] Fernando S.M., Fan E., Rochwerg B. (2021). Lung-protective ventilation and associated outcomes and costs among patients receiving invasive mechanical ventilation in the. Chest.

[9] Qadir N., Bartz R.R., Cooter M.L. (2021). Variation in early management practices in moderate-to-severe ARDS in the United States: the Severe ARDS: Generating Evidence Study. Chest.

[10] Sjoding M.W., Gong M.N., Haas C.F., Iwashyna T.J. (2019). Evaluating delivery of low tidal volume ventilation in six ICUs using electronic health record data. Crit Care Med.

[11] Harris S., Shi S., Brealey D. (2018). Critical Care Health Informatics Collaborative (CCHIC): data, tools and methods for reproducible research: a multi-centre UK intensive care database. Int J Med Inform.

[12] Pollard T.J., Johnson A.E.W., Raffa J.D., Celi L.A., Mark R.G., Badawi O. (2018). The eICU collaborative research database, a freely available multi-center database for critical care research. Sci Data.

[13] Hart M.C., Orzalesi M.M., Cook C.D. (1963). Relation between anatomic respiratory dead space and body size and lung volume. J Appl Physiol.

[14] Rubenfeld G.D., Caldwell E., Peabody E. (2005). Incidence and outcomes of acute lung injury. N Engl J Med.

[15] McNamee J.J., Gillies M.A., Barrett N.A. (2017). pRotective vEntilation with veno-venouS lung assisT in respiratory failure: a protocol for a multicentre randomised controlled trial of extracorporeal carbon dioxide removal in patients with acute hypoxaemic respiratory failure. J Intensive Care Soc.

[16] Bellani G., Laffey J.G., Pham T. (2016). Epidemiology, patterns of care, and mortality for patients with acute respiratory distress syndrome in intensive care units in 50 countries. JAMA.

[17] Neto A.S., Barbas C.S.V., Simonis F.D. (2016). Epidemiological characteristics, practice of ventilation, and clinical outcome in patients at risk of acute respiratory distress syndrome in intensive care units from 16 countries (PRoVENT): an international, multicentre, prospective study. Lancet Respir Med.

[18] Pisani L., Algera A.G., Neto A.S. (2022). Geoeconomic variations in epidemiology, ventilation management, and outcomes in invasively ventilated intensive care unit patients without acute respiratory distress syndrome: a pooled analysis of four observational studies. Lancet Glob Heal.

[19] Poole J., McDowell C., Lall R. (2017). Individual patient data analysis of tidal volumes used in three large randomized control trials involving patients with acute respiratory distress syndrome. Br J Anaesth.

[20] Laffey J.G., Madotto F., Bellani G. (2017). Geo-economic variations in epidemiology, patterns of care, and outcomes in patients with acute respiratory distress syndrome: insights from the LUNG SAFE prospective cohort study. Lancet Respir Med.

[21] Wanderer J.P., Ehrenfeld J.M., Epstein R.H. (2015). Temporal trends and current practice patterns for intraoperative ventilation at U.S. academic medical centers: a retrospective study. BMC Anesthesiol.

[22] Schwede M., Lee R.Y., Zhuo H. (2020). Clinician recognition of the acute respiratory distress syndrome: risk factors for under-recognition and trends over time. Crit Care Med.

[23] Mehta A., Dernoncourt F., Walkey A., Data M.I.T.C. (2016). Secondary Analysis of Electronic Health Records.

[24] Needham D.M., Yang T., Dinglas V.D. (2015). Timing of low tidal volume ventilation and intensive care unit mortality in acute respiratory distress syndrome. A prospective cohort study. Am J Respir Crit Care Med.

[25] Mikkelsen M.E., Dedhiya P.M., Kalhan R., Gallop R.J., Lanken P.N., Fuchs B.D. (2008). Potential reasons why physicians underuse lung-protective ventilation: a retrospective cohort study using physician documentation. Respir Care.

[26] Rubenfeld G.D., Cooper C., Carter G., Thompson B.T., Hudson L.D. (2004). Barriers to providing lung-protective ventilation to patients with acute lung injury. Crit Care Med.

[27] McNicholas B.A., Madotto F., Pham T. (2019). Demographics, management and outcome of females and males with acute respiratory distress syndrome in the LUNG SAFE prospective cohort study. Eur Respir J.

[28] Nijbroek S.G., Hol L., Swart P. (2021). Sex difference and intra-operative tidal volume: insights from the LAS VEGAS study. Eur J Anaesthesiol.

