# Supplementary material for: Low Tidal Volume Ventilation Is Poorly Implemented for Patients in North American and United Kingdom ICUs Using Electronic Health Records
Source: Chest. 2023 Sep 27;165(2):333–47. doi: 10.1016/j.chest.2023.09.021 (PMC10851261; doi:10.1016/j.chest.2023.09.021)

**Low Tidal Volume Ventilation is Poorly Implemented for Patients in North American and United Kingdom Intensive Care Units using Electronic Health Records**

**Supplementary Material**

Table of Contents

[1. e-Appendix 1. Ethical approvals 2](#_Toc149653679)

[2. e-Appendix 2. Code for extraction of ventilation and blood gas data from eICU-CRD. 3](#_Toc149653680)

[3. Supplemental tables 13](#_Toc149653681)

[Table E1. Association between sex and outcomes at 30 days for patients mechanically ventilated for greater than 48 hours from both databases. 13](#_Toc149653682)

[4. Supplemental figures 14](#_Toc149653683)

[Figure E1 Cumulative density plot of threshold V_T_ for 6 mLkg^-1^ PBW 14](#_Toc149653684)

# e-Appendix 1. Ethical approvals

Eleven intensive care units from five UK biomedical centres contribute to the CCHIC. Data sharing agreements are in place between participating centres, and 265 physiological, biochemical, demographic, diagnostic and therapeutic parameters are routinely collected. The CCHIC database was approved by the National Research Ethics Service (14/LO/1031). The IDHS has been assessed as meeting the NHS Information Governance Toolkit Level 2 and ISO 27001 standard to ensure that only deidentified data can leave the IDHS. The eICU-CRD data has been made publicly available by a collaboration between the MIT LCP and Phillips Healthcare. The eICU-CRD is fully anonymised and exempt from the requirement for further approvals as it has been certified as meeting safe harbour standards by Privacert (Cambridge, MA) (Health Insurance Portability and Accountability Act Certification no. 1031219–2).

# e-Appendix 2. Code for extraction of ventilation and blood gas data from eICU-CRD.

The attached code is written for R version 3.6.2 (R Core Team, Vienna).

An instance of the eICU-CRD needs to be created either locally or in the cloud. Instructions on how to do this, once credentialled access has been obtained, are available on the MIT LCP Physionet website (<https://physionet.org/content/eicu-crd/2.0/>).

Code for extraction of fields from CCHIC has not been provided as these data are stored securely in a data safe haven at University College London, University of London, London, UK. The same principles and cleaning steps were undertaken as for the eICU-CRD database.

|  | ## ---------------------------  ##  ## Script to extract and clean blood gas values and ventilator ## fields from the eICU-CRD  ##  ## Author: ####  ##  ## Date Created: ####  ##  ## Email: ####  ##  ## ---------------------------  # libraries and custom functions ------------------------------------------  library(dplyr)  library(tidyr)  library(RPostgres)  # database connection  ctn <- dbConnect(  RPostgres::Postgres(),  host = '####,  port = ####,  user = '******',  password = '******',  dbname = 'eicu'  )  mean_tv <- function(a, b, c, na.rm = T) {  mean(c(a, b, c), na.rm = TRUE)  }  # extract and clean tidal volume ------------------------------------------  table.eicu.respiratorycharting <-  dplyr::tbl(ctn, dplyr::sql("SELECT * FROM respiratorycharting")) %>%  dplyr::select(-respcharttypecat, -respchartentryoffset, -respchartid) %>%  collect() %>%  tbl_df()  vent_data_labels = c(  'FiO2',  "FIO2 (%)",  "Mean Airway Pressure",  "Vent Rate",  "Tidal Volume (set)",  "TV/kg IBW",  'Tidal Volume Observed (VT)',  'PEEP',  "Plateau Pressure",  "Pressure Support",  'Total RR',  "Total RR",  "Pressure Control",  "Peak Insp. Pressure",  "RR (patient)",  "Total RR",  "Mechanical Ventilator Mode",  "Exhaled MV",  "Exhaled TV (machine)",  "Exhaled TV (patient)",  "Vent Rate",  "Peak Pressure",  "Mechanical Ventilator Mode"  )  vent_obs <-  filter(table.eicu.respiratorycharting,  respchartvaluelabel %in% vent_data_labels) %>%  group_by(patientunitstayid) %>%  dplyr::arrange(respchartoffset, .by_group = TRUE)  TV_fields <- c(  'Exhaled TV (machine)',  'Exhaled TV (patient)',  'Tidal Volume Observed (VT)',  'Set Vt (Servo,LTV)',  "Set Vt (Drager)"  )  # except for a few aberrant values, most of these are in L, except for those > 150  # which are likely in ml and distributed in the same way as those < 1.  # extract and clean tidal volumes  tidal_volumes <-  table.eicu.respiratorycharting %>%  filter(respchartvaluelabel %in% TV_fields) %>%  mutate(respchartvalue = as.numeric(respchartvalue)) %>%  mutate(  respchartvalue = case_when(    (respchartvaluelabel == "Exhaled TV (machine)") &  (respchartvalue <= 0.15) ~ NA_real_,  (respchartvaluelabel == "Exhaled TV (machine)") &  (respchartvalue > 1) & (respchartvalue < 150) ~ NA_real_,  (respchartvaluelabel == "Exhaled TV (machine)") &  (respchartvalue <= 1) &  (respchartvalue >= 0.15) ~ respchartvalue * 1000,  (respchartvaluelabel == "Exhaled TV (machine)") &  (respchartvalue >= 1377) ~ NA_real_,  (respchartvaluelabel == "Exhaled TV (machine)") &  (respchartvalue > 150) &  (respchartvalue < 1377) ~ respchartvalue,  (respchartvaluelabel == "Exhaled TV (machine)") ~ respchartvalue,    (respchartvaluelabel == "Exhaled TV (patient)") &  (respchartvalue <= 0.15) ~ NA_real_,  (respchartvaluelabel == "Exhaled TV (patient)") &  (respchartvalue > 2) & (respchartvalue < 150) ~ NA_real_,  (respchartvaluelabel == "Exhaled TV (patient)") &  (respchartvalue <= 2) &  (respchartvalue >= 0.15) ~ respchartvalue * 1000,  (respchartvaluelabel == "Exhaled TV (patient)") &  (respchartvalue >= 1377) ~ NA_real_,  (respchartvaluelabel == "Exhaled TV (patient)") &  (respchartvalue >= 150) &  (respchartvalue < 1377) ~ respchartvalue,  (respchartvaluelabel == "Exhaled TV (patient)") ~ respchartvalue,    (respchartvaluelabel == "Tidal Volume Observed (VT)") &  (respchartvalue <= 1) & (respchartvalue >= 0) ~ NA_real_,  (respchartvaluelabel == "Tidal Volume Observed (VT)") &  (respchartvalue > 1) & (respchartvalue < 150) ~ NA_real_,  (respchartvaluelabel == "Tidal Volume Observed (VT)") &  (respchartvalue >= 150) &  (respchartvalue < 1377) ~ respchartvalue,  (respchartvaluelabel == "Tidal Volume Observed (VT)") &  (respchartvalue >= 1377) ~ NA_real_,  (respchartvaluelabel == "Tidal Volume Observed (VT)") ~ respchartvalue,  FALSE ~ respchartvalue  )  ) %>%  filter(!is.na(respchartvalue))  tv_clean <-  tidal_volumes %>% filter(respchartvaluelabel == "Exhaled TV (patient)") %>%  filter(!is.na(respchartvalue)) %>%  dplyr::rename(Ex_tv_patient = respchartvalue) %>%  dplyr::select(-respchartvaluelabel) %>%  full_join(  tidal_volumes %>% filter(respchartvaluelabel == "Exhaled TV (machine)") %>%  filter(!is.na(respchartvalue)) %>%  dplyr::select(-respchartvaluelabel) %>%  dplyr::rename(Ex_tv_machine = respchartvalue),  by = c('patientunitstayid', 'respchartoffset')  ) %>%  bind_rows(  tidal_volumes %>% filter(respchartvaluelabel == "Tidal Volume Observed (VT)") %>%  filter(!is.na(respchartvalue)) %>%  dplyr::rename(tv_observed = respchartvalue) %>%  dplyr::select(-respchartvaluelabel)  ) %>%  mutate(TV = mapply(  mean_tv,  Ex_tv_patient,  Ex_tv_machine,  tv_observed  )) %>%  dplyr::select(-c(Ex_tv_patient, Ex_tv_machine, tv_observed))  rm(tidal_volumes, TV_fields)  # the offest table has the start times of ventilatiuon in the epoch of the database so that values can be  # related to other measures (e.g. labs data)  offset_table <-  ungroup(tv_clean) %>%  group_by(patientunitstayid) %>%  dplyr::arrange(respchartoffset, .by_group = TRUE) %>%  slice(1) %>%  dplyr::select(patientunitstayid, respchartoffset) %>%  dplyr::rename(start_offset = respchartoffset) %>% ungroup()  tv_clean <-  tv_clean %>%  ungroup() %>%  left_join(offset_table, by = 'patientunitstayid') %>%  mutate(timer = respchartoffset - start_offset) %>%  dplyr::select(-start_offset) %>%  group_by(patientunitstayid) %>%  arrange(timer, .by_group = T) %>%  ungroup()  # list of patients ventilated < 48 hours  short_pats <-  tv_clean %>%  group_by(patientunitstayid) %>%  dplyr::arrange(desc(timer), .by_group = T) %>%  dplyr::slice(1) %>%  filter(timer < 48 * 60) %>%  dplyr::select(patientunitstayid)  tv_clean <-  filter(tv_clean,  !patientunitstayid %in% short_pats$patientunitstayid)  # work out ventilation periods by looking for >24 hour gaps in recordings  d <- list()  p <- progress_estimated(length(unique(tv_clean$patientunitstayid)))  for (pt in seq_along(unique(tv_clean$patientunitstayid))) {  p$tick()$print()  a <-  filter(tv_clean,  patientunitstayid == unique(tv_clean$patientunitstayid)[pt]) %>%  mutate (l_timer = lag(timer, default = 0)) %>%  mutate(delta_t = timer - l_timer)    b <- list()  j <- 1  for (i in 1:nrow(a)) {  if (a$delta_t[i] <= 1440) {  j <- j  } else  ( j <- j + 1 )  b[[i]] <- j  }  a$vent_period <- unlist(b)  a <- dplyr::select(a,-c(l_timer, delta_t))  d[[pt]] <- a  }# about 3 minutes  tv_clean_with_vent_periods <- bind_rows(d)  #  rm(a, b, d, j, i, p, short_pats)  # in order to account for patients who have high recording frequencies  # we need to bin the times to hour slots, we don't want to unnecessarily impute  # low recording freq patients, but we should downsample those who have minute by minute recordings  clean_tv <-  tv_clean_with_vent_periods %>%  ungroup %>%  mutate(bin = floor(timer / 60)) %>%  mutate(kvp = paste(patientunitstayid, bin, sep = "_")) %>%  group_by(kvp) %>%  summarise(binned_tv = median(TV, na.rm = T)) %>%  right_join(  tv_clean_with_vent_periods %>%  mutate(bin = floor(timer / 60)) %>%  mutate(kvp = paste(patientunitstayid, bin, sep = "_")) %>%  group_by(kvp) %>%  slice(1),  by = "kvp"  ) %>%  dplyr::select(-kvp) %>%  dplyr::select(everything(), binned_tv)  short_vents_2 <-  clean_tv %>% group_by(patientunitstayid, vent_period) %>% # short initial ventilator episodes, often preceeding admission  mutate(index = n()) %>% filter((index< 5) & (vent_period == 1)) %>%  mutate(kvp = paste(patientunitstayid, vent_period, sep= "_"))  clean_tv <-  clean_tv %>%  mutate(kvp = paste(patientunitstayid, vent_period, sep= "_")) %>%  filter(!kvp %in% short_vents_2$kvp) %>% #762,189  dplyr::select(-kvp) #762,165 x 7  rm(short_vents_2)  # Extract and clean FiO2 --------------------------------------------------  fi_fields <- c("FiO2", "FIO2 (%)", "Set Fraction of Inspired Oxygen (FIO2)")  vent_fi <-  table.eicu.respiratorycharting %>%  filter(patientunitstayid %in% clean_tv$patientunitstayid) %>%  filter(respchartvaluelabel %in% fi_fields) %>%  mutate(respchartvalue = gsub(pattern = "%", replacement = "", respchartvalue)) %>%  mutate(respchartvalue = as.numeric(respchartvalue) ) %>%  mutate(respchartvalue = case_when(  (respchartvalue <= -21) ~ respchartvalue*(-1),  (respchartvalue <= 0) & (respchartvalue >= -20) ~ NA_real_,  (respchartvalue <= 0.2) & (respchartvalue >= 0) ~ NA_real_,  (respchartvalue >= 0.21) & (respchartvalue <= 1) ~ respchartvalue * 100,  (respchartvalue > 1) & (respchartvalue <= 20) ~ NA_real_,  (respchartvalue >= 21 ) & (respchartvalue <= 100) ~ respchartvalue,  (respchartvalue > 100) & (respchartvalue <= 209) ~ NA_real_,  (respchartvalue >= 210) & (respchartvalue <= 1000) ~ respchartvalue/10,  (respchartvalue >= 1000) & (respchartvalue <= 2099) ~ NA_real_,  (respchartvalue >= 2100) & (respchartvalue <= 10000) ~ respchartvalue/100,  respchartvalue > 10000 ~ NA_real_,  FALSE ~ respchartvalue)) %>%  filter(!is.na(respchartvalue)) %>%  left_join( offset_table, by = 'patientunitstayid') %>%  mutate(timer = respchartoffset - start_offset) %>%  dplyr::select(- start_offset) %>%  group_by(patientunitstayid) %>%  arrange(timer, .by_group = T) %>%  ungroup() %>%  dplyr::select(-respchartvaluelabel) %>%  mutate(bins = floor(timer / 60))  vent_fi <- vent_fi %>%  mutate(kvp = paste(patientunitstayid, bins, sep = "_")) %>%  group_by(kvp) %>%  summarise(FiO2 = mean(respchartvalue, na.rm = T)) %>% # averages FiO2 if more than 1 reading / hr  left_join(vent_fi %>%  mutate(kvp = paste(patientunitstayid, bins, sep = "_")) %>%  group_by(kvp) %>% slice(1),by = 'kvp' ) %>%  dplyr::select(-kvp)  rm(fi_fields)  # Extract and clean PEEP --------------------------------------------------  peep_fields <- c("PEEP", "PEEP/CPAP")  vent_peep <-  table.eicu.respiratorycharting %>%  filter(patientunitstayid %in% clean_tv$patientunitstayid) %>%  filter(respchartvaluelabel %in% peep_fields) %>%  mutate(respchartvalue = as.numeric(respchartvalue)) %>%  filter(!is.na(respchartvalue)) %>%  mutate(respchartvalue = case_when(  respchartvalue > 40 ~ NA_real_,  respchartvalue < 0 ~ NA_real_,  (respchartvalue >=0) & (respchartvalue <= 40) ~ respchartvalue,  FALSE ~ respchartvalue)) %>%  filter(!is.na(respchartvalue)) %>%  left_join( offset_table, by = 'patientunitstayid') %>%  mutate(timer = respchartoffset - start_offset) %>%  dplyr::select(- start_offset) %>%  group_by(patientunitstayid) %>%  arrange(timer, .by_group = T) %>%  ungroup() %>%  dplyr::select(-respchartvaluelabel) %>%  mutate(bins = floor(timer / 60))  vent_peep <- vent_peep %>%  mutate(kvp = paste(patientunitstayid, bins, sep = "_")) %>%  group_by(kvp) %>%  summarise(PEEP = mean(respchartvalue, na.rm = T)) %>%  right_join(vent_peep %>%  mutate(kvp = paste(patientunitstayid, bins, sep = "_")) %>%  group_by(kvp) %>% slice(1), by = 'kvp' ) %>%  dplyr::select(-kvp)  rm(peep_fields)  # Retrieve and clean ABGs -------------------------------------------------  gas_fields <-  c("paO2", "paCO2", "pH", "FiO2", "Temperature", "O2 Sat (%)")  gas_columns <-  c(  "patientunitstayid",  "labresultrevisedoffset",  "labname",  "labresulttext",  "labresult"  )  gases <- dplyr::tbl(ctn, "lab") %>%  filter(labtypeid == 7) %>%  dplyr::select(gas_columns) %>%  filter(labname %in% gas_fields) %>%  group_by(patientunitstayid) %>%  dplyr::arrange(labresultrevisedoffset, .by_group = TRUE) %>%  dplyr::mutate(kvp = paste(patientunitstayid, labresultrevisedoffset, labname, sep = "-")) %>%  collect() %>%  mutate(labresulttext = gsub(pattern = " ", replacement = "", labresulttext)) %>% # remove spaces,  mutate(labresulttext = gsub(pattern = "<", replacement = "", labresulttext)) %>% # remove < character  mutate(labresulttext = gsub(pattern = ">", replacement = "", labresulttext)) %>% # remove < character  mutate(labresulttext = gsub(pattern = "%", replacement = "", labresulttext)) # remove % markers  duplicated_gases <-  gases %>%  ungroup() %>%  group_by(kvp) %>%  slice(1) %>% ungroup() %>%  dplyr::select(-kvp,-labresult) %>%  gather(key,  value,  -patientunitstayid,  -labresultrevisedoffset,  -labname) %>%  dplyr::select(-key) %>%  tidyr::spread(labname, value)  gases_step_2 <-  gases %>%  filter(!duplicated(kvp)) %>%  dplyr::select(-kvp, -labresult) %>% ungroup() %>%  gather(key, value, -patientunitstayid, -labresultrevisedoffset,-labname) %>%  dplyr::select(-key) %>%  tidyr::spread(labname, value) %>%  bind_rows(duplicated_gases) %>%  arrange(patientunitstayid, labresultrevisedoffset) %>%  dplyr::rename(SaO2 = `O2 Sat (%)`) %>%  mutate(paO2 = as.numeric(paO2)) %>%  mutate(SaO2 = as.numeric(SaO2)) %>%  mutate(paCO2 = as.numeric(paCO2)) %>%  mutate(FiO2 = as.numeric(FiO2)) %>%  mutate(SaO2 = case_when(  SaO2 < 0 ~ NA_real_,  (SaO2 > 100) & (SaO2 <= 105) ~ 100,  SaO2 > 900 ~ SaO2 / 100,  SaO2 > 106 ~ NA_real_,  (SaO2 <= 100) & (SaO2 > 0) ~ SaO2,  FALSE ~ NA_real_  )) %>%  mutate(FiO2 = case_when(  (FiO2 > 0.2 ) & (FiO2 <= 1) ~ FiO2 * 100,  FiO2 < -1 ~ FiO2 * -1,  (FiO2 < 0.2 ) & (FiO2 -1) ~ NA_real_,  FiO2 > 100 ~ NA_real_,  (FiO2 > 1) & (FiO2 <= 20) ~ NA_real_,  (FiO2 > 20) & (FiO2 <= 100) ~ FiO2,  FALSE ~ NA_real_)) %>%  mutate(kvp = paste(patientunitstayid, labresultrevisedoffset)) %>%  dplyr::filter(!duplicated(kvp)) %>%  dplyr::select(-kvp)  rm(duplicated_gases)  #The FiO2 and gas data don't quite align, need to make bins for time points  #for each gas and find all the FiO2s in each common bin.  gases_step_3 <-  gases_step_2 %>%  filter(!is.na(paO2)) %>%  mutate(bin_hour = ifelse(  (labresultrevisedoffset %% 120 < 90),  floor((labresultrevisedoffset + 30) / 120) ,  floor(labresultrevisedoffset / 120)  )) %>%  mutate(kvp = paste(patientunitstayid, bin_hour)) %>%  dplyr::select(-FiO2) %>%  left_join(  gases_step_2 %>%  filter(!is.na(FiO2)) %>%  mutate(bin_hour = ifelse(  (labresultrevisedoffset %% 120 < 90),  floor((labresultrevisedoffset + 30) / 120),  floor(labresultrevisedoffset / 120)  )) %>%  mutate(kvp = paste(patientunitstayid, bin_hour)) %>%  dplyr::select(FiO2, kvp),  by = 'kvp'  ) %>%  dplyr::select(-kvp,-bin_hour) %>%  dplyr::select(patientunitstayid, labresultrevisedoffset, FiO2, everything())  rm(gas_fields, gas_columns, gases, gases_step_2) |  |
| --- | --- | --- |

# Supplemental tables

Table E1. Association between sex and outcomes at 30 days for patients mechanically ventilated for greater than 48 hours from both databases.

Outcomes at day 30 were assessed using competing outcomes and expressed as sub-distribution hazards. For example, with respect to the outcome of being extubated by day 30, death was used as a competing hazard. Given the observed associations between height and adherence with LTVV (Table 2), and between LTVV and outcome (Table 4), we wanted to ensure that we were not unmasking sex differences in outcomes by focusing on adherence to LTVV.

|  | **CCHIC** | |  | **eICU-CRD** | |
| --- | --- | --- | --- | --- | --- |
|  | **SHR (95% CI)** | ***P*** |  | **SHR (95% CI)** | ***P*** |
| **Outcome: Death** | | | | | |
| Sex: male | 0.95  (0.95-1.13) | 0.43 |  | 0.95  (0.86 -1.06) | 0.39 |
| Age | 1.01  (1.01-1.01) | <0.001 |  | 1.01  (1.01-1.01) | <0.001 |
| APACHE | 1.06  (1.05-1.07) | <0.001 |  | 1.02  (1.01-1.02) | <0.001 |
| **Outcome: Extubation** | | | | | |
| Sex: male | 0.94  (0.95-1.13) | 0.42 |  | 0.93  (0.89-0.98) | 0.01 |
| Age | 0.99  (0.994-0.999) | 0.01 |  | 0.99  (0.94-1.04) | 0.36 |
| APACHE | 0.94  (0.93-0.95) | <0.001 |  | 0.98  (0.93-1.03) | 0.55 |

SHR: Cause-specific hazard ratio; AHRF: Acute hypoxic respiratory failure; PBW: Predicted body weight.

# Supplemental figures

## Figure E1 Cumulative density plot of threshold V_T_ for 6 mLkg^-1^ PBW

Cumulative distribution plot demonstrating the percentage of patients that would be in receipt of > 6 mlkg^-1^ PBW tidal volumes at increasing tidal volumes based on the height and gender of patients in each database. In order to reduce the risk of patients receiving > 6 mlkg^-1^ PBW to less than 50%, starting tidal volumes should be set to 322ml for female and 426ml for male patients.


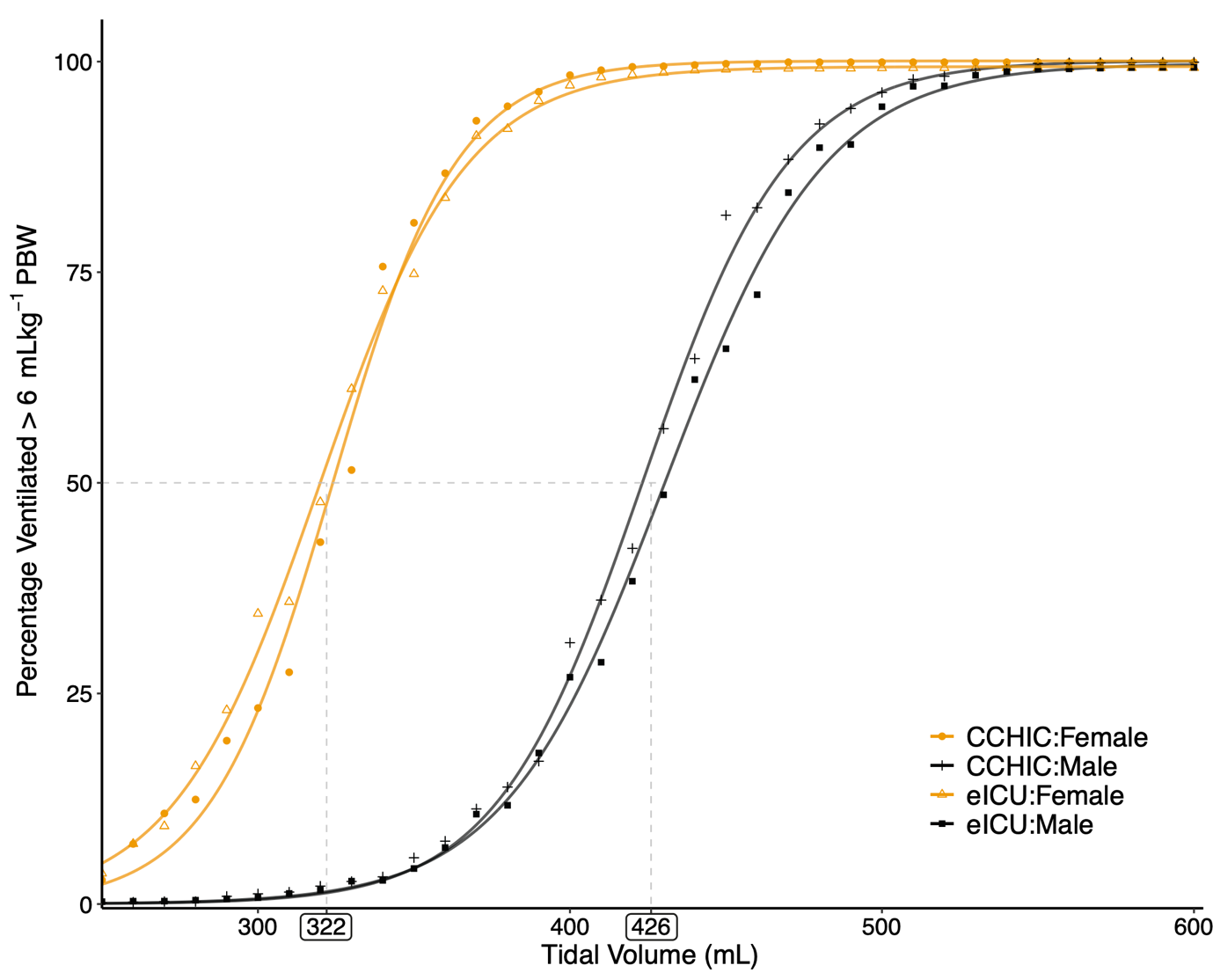

Supplement: e-Online Data [file mmc1.docx]
